# Two vacuolar invertase inhibitors PpINHa and PpINH3 display opposite effects on fruit sugar accumulation in peach

**DOI:** 10.3389/fpls.2022.1033805

**Published:** 2022-12-14

**Authors:** Md Dulal Ali Mollah, Xian Zhang, Li Zhao, Xiaohan Jiang, Collins O. Ogutu, Qian Peng, Mohammad A. A. Belal, Qiurui Yang, Yaming Cai, Elsayed Nishawy, Sylvia Cherono, Lu Wang, Yuepeng Han

**Affiliations:** ^1^ Key Laboratory of Plant Germplasm Enhancement and Specialty Agriculture, Wuhan Botanical Garden, The Innovative Academy of Seed Design, Chinese Academy of Sciences (CAS), Wuhan, China; ^2^ Hubei Hongshan Laboratory, Wuhan, China; ^3^ University of Chinese Academy of Sciences, Beijing, ;China; ^4^ Genetic Resource Department, Egyptian Deserts Gene Bank, Desert Research Center, Cairo, Egypt; ^5^ Sino-African Joint Research Center, Chinese Academy of Sciences, Wuhan, China

**Keywords:** *Prunus persica*, soluble sugars, sugar transporter, invertase inhibitor, sucrose phosphate synthase

## Abstract

Soluble sugars are an important determinant of fruit taste, but their accumulation mechanisms remain elusive. In this study, we report two vacuolar invertase inhibitor genes involved in sugar accumulation in peach, *PpINHa* and *PpINH3*. Transient overexpression of *PpINH3* in peach fruits resulted in an increase in sugar content, while the opposite trend was detected for *PpINHa*. Unexpectedly, PpINH3 and PpINHa both had no physical interaction with vacuolar invertase (VIN). Moreover, the *PpVIN* genes had no or extremely low expression in fruits at the ripening stage. These results suggested that the regulatory role of PpINHa and PpINH3 in sugar accumulation is unlikely due to their interaction with PpVINs. Additionally, overexpression of *PpINHa* and *PpINH3* had an impact on transcription of genes related to fruit sugar metabolism and transport, which is likely responsible for their regulatory role in fruit sugar accumulation. Altogether, these results indicated an important role of *PpINHs* in fruit accumulation in peach. Our study provides new insights into molecular mechanisms underlying sugar accumulation, which could be useful for genetic improvement of fruit taste in breeding programs of peach and other fruit crops.

## 1 Introduction

Peach (*Prunus persica L*. Batsch), a diploid deciduous perennial woody species of the family *Rosaceae*, is a major economic crop worldwide, especially in the temperate zones. It is cultivated as ornamental purpose and/or fruit production. In addition, peach also serves as a model plant for comparative and functional genomic studies in the *Rosaceae* family due to its short juvenile period, self-compatibility and a small genome size of ~ 230 Mb per haploid (Verde et al., 2013). Peach consumption has showed a downward trend in recent years due to poor or inconsistent flavor quality (Cirilli et al., 2016). Thus, improvement of fruit quality is becoming one of the most important goals of peach breeding programmes.

Sweetness has a great influence on the degree of consumer satisfaction for peach fruits. Sweetness is attributed to the composition and content of soluble sugars, such as sucrose, glucose, fructose and sorbitol. Hence, soluble sugars represent a fundamental component of fruit edible quality (Colaric et al., 2005). Fruit sugar metabolism is a complex metabolic pathway that begins with the transport of photoassimilates through phloem sieve elements into the sink tissue. In the family Rosaceae including peach, sorbitol acts as the principal photosynthate and translocated sugar, in addition to sucrose, the most common translocated carbon (Zanon et al., 2015). Sorbitol enters into fruit cells upon phloem unloading, while sucrose either directly enters into fruit cells, or is hydrolyzed into glucose and fructose by cell wall invertase (CIN) that are subsequently taken up into fruit cells. The hydrolysis of sucrose can also occur in the vacuole by vacuolar invertase (VIN). In cytoplasm, sorbitol is converted into fructose and glucose by sorbitol oxidase (SOX) and sorbitol dehydrogenase (SDH), respectively. Sugar metabolic pathway in cytoplasm involves various enzymes, such as neutral invertase (NIV), fructokinase (FK), hexokinase (HK), sucrose synthase (SuSy), and sucrose phosphate synthase (SPS), producing different primary metabolites such as fructose-6-phosphate (F6P) and soluble sugars (Lombardo et al., 2011; Wang et al., 2016). Transport of soluble sugars into the vacuole is mediated by sugar transporters, such as tonoplast sugar transporters (TSTs), vacuolar glucose transporters (VGTs), and sucrose transporters (SUCs/SUT*s*) (Eom et al., 2015).

In peach, fruit sugar content not only varies throughout development, but it also shows a great variation among cultivars (Vimolmangkang et al., 2016). Overall, total sugar content shows an increasing trend during fruit development and reaches a peak at ripening stages, with sucrose being the predominant sugar and sorbitol being the minor one in cultivated peaches. SuSy and VINs are likely responsible for the predominant accumulation of sucrose in peach fruits (Vimolmangkang et al., 2016), while SDH that converts sorbitol into fructose plays an important role in determining fructose content (Kanayama et al., 2005). To investigate the genetic basis of fruit sugar accumulation in peach, quantitative trait locus (QTL) mapping has been extensively conducted and QTLs for fruit sugar content have been identified on nearly all chromosomes (Chrs) (Dirlewanger et al., 1999; Etienne et al., 2002; Dirlewanger et al., 2004; Quilot et al., 2005; Illa et al., 2011; Salazar et al., 2014; Zeballos et al., 2016; Mora et al., 2017; Font I Forcada et al., 2018). However, few candidate genes for fruit sugar content have been identified. A recent functional study shows that a *TST* gene located in the QTL interval on Chr 5 is involved in the regulation of fruit sugar accumulation in peach (Peng et al., 2020), which was confirmed in a later study (Yu et al., 2021). The important roles of TSTs in fruit sugar accumulation have been also demonstrated using forward and reverse genetic approaches in many other crops, such as sugar beet (Jung et al., 2015), watermelon (Ren et al., 2018), *Cucumis melo* (Cheng et al., 2018), apple and tomato (Ma et al., 2017; Zhu et al., 2021). Nevertheless, the complete mechanism underlying fruit sugar accumulation is yet to be elucidated in peach.

For the complex trait of fruit organoleptic quality, transcriptome sequencing has been used to identify candidate genes responsible for sugar content, which provides a basis for understanding molecular mechanism regulating sugar metabolism and accumulation (Aslam et al., 2019). The availability of the high-quality draft genome of the doubled haploid peach cv. ‘Lovell’ has facilitated identification of candidate genes controlling various metabolic pathways in peach (Verde et al., 2013). In this study, the profiles of fruit sugar accumulation in four cultivars and one wild relative *Prunus davidiana* were investigated and candidate genes for fruit sugar accumulation were also identified using comparative transcriptome analysis. Our results are helpful for understanding the mechanism of fruit sugar accumulation in peach and other fruit crops.

## 2 Materials and methods

### 2.1 Plant materials

All peach accessions used in this study, including two yellow-fleshed and freestone nectarine cultivars, Ligelante (LG) and Meiguowanyou (MY), two white-fleshed and clingstone peach cultivars Xiacui (XC) and Xiahui (XH), and one wild relative Shantao (*P. davidiana*), are maintained in the orchard of the Northwest A & F University, Shaanxi Province, China. All the four cultivars tested were grafted on Shantao rootstock and have similar ripening periods. Peach trees were planted at a spacing of 4 m × 1 m and grown under standard conventional field practices, including irrigation, fertilization and pest control. Fruit samples were collected at 34, 75 and 117 days after full bloom (DAF), which corresponded to the first exponential growth stage, the second exponential growth stage and the ripening stage, respectively. Three biological replicates were conducted for each treatment and each replicate contained 6-8 fruits. Fruit sample were peeled, cut into small pieces, frozen in liquid nitrogen, and then stored at -80°C for use.

### 2.2 Measurement of fruit sugar content and composition

Fruit sugar extraction was conducted according to our previous study (Vimolmangkang et al., 2016). Measurement of sugar type and content was performed using high-performance liquid chromatography (HPLC) following the previously reported protocol (Ma et al., 2015). Briefly, the sugar contents of fruit samples were measured using an Agilent 1260 Infinity HPLC system (Milford, MA, USA) equipped with a refractive index detector (Shodex RI-101; Shodex Munich, Germany). A Transgenomic COREGET-87C column (7.8 mm × 300 mm, 10 μm) with a guard column (Transgenomic CARB Sep Coregel 87C) was used to perform separation and column temperature was maintained at 85°C by a Dionex TCC-100 thermostated column compartment. Flow rate at the mobile phases were maintained at the rate of 0.6 mL/min with degassed, distilled, and deionized water. Sugar concentrations were expressed on a fresh weight (FW) basis. Total sugar content was specified by the amount of four sugar components, sucrose, fructose, glucose, and sorbitol.

### 2.3 RNA extraction, RNA-Seq library construction and sequencing

Fruit samples were ground into powder and then subjected to total RNA extraction using EASYspin Plus Plant RNA Mini Kit (Aidlab, Beijing, China) according to the manufacturer’s instructions. RNA concentration and purity were checked with the ND-1000 UV–Vis spectrophotometer (Nanodrop^®^, http://www.nanodrop.com/), while RNA integrity was evaluated with the RNA Nano 600 Assay Kit of the Bioanalyzer 2100 system (Agilent Technologies, CA, USA).

NEBNext^®^ Ultra ™ RNA Library Prep Kit for Illumina^®^ (NEB, USA) were used to construct the RNA sequencing libraries according to the manufacturer’s instruction. The quality of these libraries was also checked through Agilent Bioanalyzer 2100 system. The index-coded samples were clustered using the HiSeq PE Cluster Kit v4-cBot-HS (Illumina) according to manufacturer’s instruction. After cluster generation, the libraries were sequenced on an Illumina sequencing platform with a paired-end 150-bp sequencing strategy.

### 2.4 Data process and differential gene expression analysis

Adaptor sequences, empty reads and low-quality reads were trimmed from raw data, with quality scores less than Q30. The high-quality clean reads were aligned to the reference genome of peach (Verde et al., 2013) using HISAT2 (Kim et al., 2015), and the index of the reference genome was built using Bowtie2.0.6 (Langmead et al., 2009). The number of clean reads mapped to each gene was counted using the HTSeq v0.6.0 software. The expression level of each gene was calculated according to the value of expected number of fragments per kilobase of transcript sequence per millions base pairs sequenced (FPKM). The read counts between samples were standardized through scaling the number of reads in a given library to a common value across all sequenced libraries using the edgeR software (version 2.6.10) (Robinson et al., 2010). In the comparing data pairs, the genes were firstly filtered manually with one FPKM value > 0.1 in at least one sample. Differentially expressed genes (DEGs) were identified using the program DESeq2 (Love et al., 2014). *P*-value was adjusted by the Benjamini and Hochberg approach. Genes with a threshold false discovery rate (FDR) ≤ 0.05 and a log2-fold change ≥ 1 were defined as DEGs. The intersections of DEGs were calculated using the online Draw Venn Diagram program (http://bioinformatics.psb.ugent.be/webtools/Venn/). Pearson correlation coefficient was used to estimate whether there was a significant relationship between gene expression levels and sugar contents.

### 2.5 Gene ontology visualization and enrichment analysis

Gene Ontology (GO) annotation results of DEGs were visualized and compared with the online program WEGO (Web Gene Ontology Annotation Plot, http://wego.genomics.cn/). GO enrichment of DEGs was implemented using Cytoscape software version 3.8.0 (Shannon et al., 2003) as well as its plugin BiNGO version 3.0.4 (Maere et al., 2005) with default the parameters. Gene networks were visualized using BiNGO.

### 2.6 Gene expression analysis using quantitative real-time RT-PCR

Total RNA was extracted using the Universal Plant Total RNA Extraction Kit (BioTeke, Beijing, China) according to the manufacturer’s instructions. The RNA samples were treated with DNase I (Takara, Dalian, China), and then subjected to cDNA synthesis with cDNA Synthesis Kit (VAZYME Biotech co. Ltd., Nanjing, China) according to the manufacturer’s instructions. qRT-PCR was conducted in a total volume of 20 µL reaction, which contained 10 µL of 2X Hieff^®^ qPCR SYBR Green Master Mix (High Rox) reagent (YEASEN biotech coo. Ltd., Shanghai, China), 2.0 µL cDNA sample and 0.4 µL of each primer (0.2 µM). qRT-PCR was performed using the Applied Biosystems stepone plus Real-Time PCR System (Applied Biosystems, USA) and the program was as follows: 5 min at 95°C followed by 40 cycles of heating at 95°C for 10 s, with annealing temperature of 60°C for 30 S. The peach gene *eIF-4E* was used as the internal control. All analyses consisted of three biological replications. Gene expression level was quantified using the 2^-ΔΔCT^ method (Livak and Schmittgen, 2001). Primer sequences used for qRT-PCR analysis are listed in **Table S1**.

### 2.7 Gene functional analysis by transient overexpression in peach fruits

Total RNA extraction and cDNA synthesis was conducted following the same protocol as described above. The full-length coding sequence was introduced into pSAK277 and then transformed into the *A. tumefaciens* strain GV3101. The transformed *Agrobacterium* was cultured at 28°C until OD_600nm_ reached to the range of 1.5 ~ 2.0. *Agrobacterium* was harvested by centrifuge at 1,000g for 5min. The precipitate was resuspended using the infiltration buffer that contained 10 mM 2-(N-morpholino) ethanesulphonic acid (MES), 10 mM MgCl_2_ and 150 μM acetosyringone, and was adjusted to pH5.6 with 1M NaOH. The concentration of *Agrobacterium* was diluted to an OD_600nm_ value of approximately 0.4 and used to infiltrate peach fruits of ‘Xia Huanjin’ at the ripening stage using our previously reported protocol (Peng et al., 2020). *Agrobacterium* cultures carrying candidate gene was injected into one side of the fruit, while the opposite side infiltrated with *Agrobacterium* cultures carrying the empty vector was used as control. At least three biological replications were carried out for each treatment, and sample collection was performed 5 days after infiltration.

### 2.8 RNA-seq data from the fruits of peach cultivars

Fruit transcriptome data for all tested cultivars were retrieved from our previous study, including two stages of 75 and 117 DAFB with three biological replicates for each stage (Zheng et al., 2021).

## 3 Results

### 3.1 Difference in fruit sugar accumulation between cultivated and wild peaches

**Figure 1:**
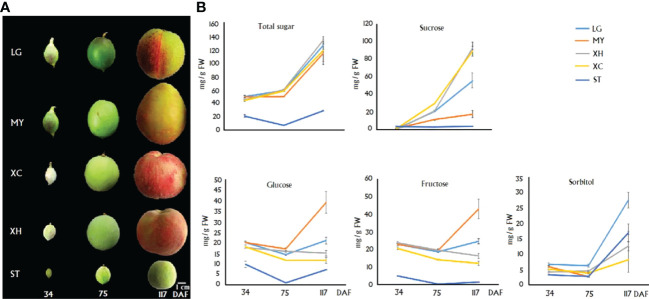
Graphical presentation of fruits at three differenet development stages. **(A)** and the correspoding sugar contents **(B)** in four cultivars and a wild relative ST. Total sugar represents the sum of glucose, fructose, sucrose and sorbitol. Error bars indicate the standard error (SE) of three biological replicates.

The contents of sugar components in fruits of four cultivars, LG, MY, XC and XH, and one wild relative Shantao (ST), at 34, 75 and 117 DAF, which were designated S1, S2 and S3, respectively (**Figure 1A**), were measured. Total sugar content showed an increasing trend in cultivated fruits throughout development, while a V-shape trend was observed for ST, with the highest level at S3 (**Figure 1B**). Total sugar contents at S1 - S3 in cultivars were all significantly higher than those in ST. For three cultivars, LG, XC and XH, sucrose content had a dramatic increase during late stages of fruit development, while both glucose and fructose contents showed a relatively small variation throughout fruit development. For cultivar MY, sucrose content showed a slight increase throughout fruit development, while both glucose and fructose contents had a dramatic increase during late stages of fruit development. Accordingly, sucrose represented the predominant soluble sugar in ripen fruits of LG, XC and XH, accounting for 43%, 67% and 74% of total sugars, respectively. However, monosaccharides instead of sucrose represented the main soluble sugars in ripen fruits of MY as glucose, fructose and sucrose accounted for 34%, 37% and 15% of total sugars, respectively.

In wild relative ST, sucrose content was almost constant throughout fruit development, while a decreasing trend and a V-shape trend were observed for fructose and glucose contents, respectively. Sorbitol content showed an increase during late stages of fruit development. Accordingly, sorbitol represented the predominant sugar compound in fruits of ST at the S3 stage, accounting for 58% of total sugars, followed by glucose (25%), sucrose (13%) and fructose (4%). Sorbitol also showed a dramatic accumulation during late stages of fruit development in LG and MY, accounting for 22% and 14% of total sugars in fruits at S3, respectively. By contrast, sorbitol content displayed a slight increase throughout fruit development in two cultivars, XH and XC.

### 3.2 Identification of DEGs associated with sugar content in fruits of peach

Since total sugar contents in cultivated fruits throughout development were much higher than those in wild fruits of ST, genes differentially expressed between cultivated and wild fruits were identified using comparative transcriptome analysis. For wild relative ST, six RNA-Seq libraries were constructed from fruit samples at S2 and S3 with three biological replicates per stage and subsequently sequenced (**Table 1**). Biological replicates had high correlation coefficients of 0.99~1.0 (**Figure S1**), suggesting that the generated RNA-Seq data were suitable for differentially expressed gene (DEG) analysis. On average, each library consisted of 44.5 million clean reads, with 6.7 Gb in size. Approximately 39.4 million (88.5%) clean reads were anchored to the peach reference genome, with 38.2 million clean reads (86.5%) uniquely mapped. The RNA-seq data of the wild fruits were compared to our previously reported transcriptome data of cultivated fruits at the S2 and S3 stages (Zheng et al., 2021).

**Table 1:** Summary of the clean RNA-Seq reads for each treatment*.

Sample	No. of total reads	Size of total reads (bp)	Q30 (%)	No. of mapped reads*			Mapping rate (%)
				Unique	Multiple	Total	
ST_75_1	47,016,830	7,052,524,500	94.58	30,784,756	1,256,748	32,041,504	68.15
ST_75_2	44,531,768	6,679,765,200	93.98	39,392,054	1,406,722	40,798,776	91.62
ST_75_3	45,538,266	6,830,739,900	85.39	35,976,986	1,209,393	37,186,379	81.66
ST_117_1	43,404,190	6,510,628,500	85.74	34,753,140	907,035	35,660,175	82.16
ST_117_2	41,856,720	6,278,508,000	85.83	33,592,111	827,183	34,419,294	82.23
ST_117_3	43,069,962	6,460,494,300	85.73	34,409,946	939,735	35,349,681	82.07

*Unique and multiple indicate reads mapped to single or multiple sites of the peach genome, respectively.

**Figure 2:**
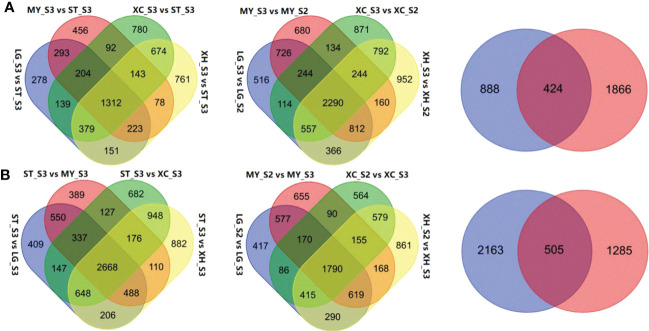
Identification of DEGs associated with total sugar content in peach fruits between different genotypes or developmental stages. **(A)**, Venn diagrams showing the numbers of DEGs up-regulated in cultivated fruits at the S3 stage relative to wild fruits (left), up-regulated in cultivated fruits at the S3 stage relative to the S2 stages (middle), the intersection of the commonly up-regulated genes across cultivars and the commonly up-regulated genes across the developmental stages (right). **(B)**, Venn diagrams showing the numbers of DEGs down-regulated in cultivated fruits at the S3 stage relative to wild fruits (left), down-regulated in cultivated fruits at the S3 stage relative to the S2 stages (middle), the intersection of the commonly down-regulated genes across cultivars and the commonly down-regulated gene across the developmental stages (right).

Comparative transcriptome analysis revealed 2,980, 2,801, 3,712, and 3,711 DEGs that were up-regulated in fruits of LG, MY, XC and XH at S3, respectively, relative to fruits at S3 of ST, with 1,312 common DEGs (**Figure 2A**). By contrast, 5,453, 4,845, 5,733, and 6,126 DEGs were down-regulated in fruits of LG, MY, XC and XH at S3, respectively, with 2,668 common DEGs (**Figure 2B**). Since total sugar content showed a significant increase in cultivated fruits during late stages of fruit development, genes differentially expressed between the S2 and S3 stages were investigated. As a result, 5,625, 5,290, 5,246, and 6,173 genes were identified to be up-regulated in fruits of LG, MY, XC and XH at S3, respectively, with 2,290 common DEGs, while 4,364, 4,224, 3,849, and 4,877 genes were down-regulated in fruits of LG, MY, XC and XH at S3, respectively, with 1,790 common DEGs. The intersection of the above 1,312 and 2,290 commonly up-regulated DEGs consisted of 424 genes (**Table S2**), whose transcript levels were positively correlated with total sugar content. By contrast, the intersection of the above 2,668 and 1,790 commonly down-regulated DEGs contained 505 genes (**Table S2**), whose transcript levels were negatively correlated with total sugar content. To validate the RNA-Seq data, the expression levels of seven genes (**Table S1**) randomly selected from the DEGs associated with sugar accumulation were validated using qRT-PCR (**Figure S2**). The qRT-PCR results of all tested genes were well consistent with those of RNA-seq data, suggesting that the results of comparative transcriptome analysis are reliable in this study.

Of the 424 up-regulated intersection genes, one (*Prupe_5G006300*) is a previously reported sugar transporter *PpTST1* that has been proven to regulate fruit sugar accumulation (Peng et al., 2020). Besides *PpTST1*, two encoded early responsive to dehydration 6-like (ERD6-Like) monosaccharide transporters, *Prupe_4G042700* and *Prupe_3G066300*, designated *PpERD6-Like1* and *PpERD6-Like2*, respectively. *PpERD6-Like1* and *PpERD6-Like2* both had similar expression patterns to *PpTST1* (**Figure S3**), and their expression levels were significantly positively correlated with total sugar content and sucrose content (**Table 2**). These results suggested that these two *PpERD6-Like* genes have putative roles in fruit sugar accumulation. Three belonged to sugar metabolic pathway genes, including two *SPS* genes, *Prupe_1G159700* and *Prupe_1G483200*, designated *PpSPS1* and *PpSPS2*, respectively, and one *SuSy* gene, *Prupe_7G192300* designated *PpSuSy1.* The expression of both *PpSPS1* and *PpSPS2* had a significant positive correlation with total sugar content and sucrose content (**Table 2**), consistent with previous reports that *PpSPS2* is good candidate for fruit sugar accumulation (Li et al., 2021). The expression of *PpSuSy1* was positively and significantly correlated with sucrose content, and positively but not significantly with total sugar content (**Table 2**). One (*Prupe_1G114500*) encoding plant invertase inhibitor, which was termed *PpINH3* but with lack of any function evidence in a recent study (Wang et al., 2020). The expression of *PpINH3* was significantly positively correlated with total sugar content and sucrose content (**Table 2**), suggesting its putative role in fruit sugar accumulation.

**Table 2:** Pearson correlation coefficients between gene expression and sugar content*.

DGE		GDR accession	Total sugar content	Sucrose content
Type	Gene name			
Up-regulated	*PpSPS1*	*Prupe_1G159700*	0.91	0.90
	*PpSPS2*	*Prupe_1G483200*	0.69	0.92
	*PpSuSy1*	*Prupe_7G192300*	0.49	0.66
	*PpTST1*	*Prupe_5G006300*	0.80	0.70
	*PpERD6-Like1*	*Prupe_4G042700*	0.85	0.74
	*PpERD6-Like2*	*Prupe_3G066300*	0.77	0.76
	*PpINH3*	*Prupe:1G114500*	0.82	0.90
Down-regulated	*PpSuSy2*	*Prupe_5G241700*	-0.72	-0.69
	*PpINHa*	*Prupe_4G001200*	-0.76	-0.74

*Pearson correlation coefficients larger than 0.64 are statistically significant at P < 0.01.

Of the 505 down-regulated intersection DEGs, one (*Prupe_5G241700*) belonged the *SuSy* gene family, designated *PpSuSy2*, and one (*Prupe_4G001200*) encoded an invertase inhibitor, designated *PpINHa*. The expression levels of *PpSuSy2* and *PpINHa* were significantly negatively correlated with total sugar content and sucrose content (**Table 2**), suggesting their potential role in fruit sugar accumulation. Notably, *PpSuSy2* and *PpINHa* both showed weak expression in cultivated fruits at the ripening stage, but with high expression in wild fruits of ST (**Figure S3**). By contrast, their homologous genes *PpSuSy1* and *PpINH3* displayed high expression in cultivated fruits at the ripening stage, but weakly expressed in wild fruits of ST (**Figure S3**).

Taken together, the above results indicated 9 candidate DEGs associated with fruit sugar accumulation, including 4 sugar metabolic pathway genes, *PpSPS1/2* and *PpSuSy1/2*, 3 sugar transporter genes, *PpTST1* and *PpERD6-Like1/2*, and 2 regulators of sugar accumulation, *PpINH3* and *PpINHa*. The homologous gene pairs *PpSuSy1/2* and *PpINH3/a* may have undergone functional divergence. Since few reports are available on the functional role of the *INH* genes in sugar accumulation, *PpINH3* and *PpINHa* were selected for further functional analysis.

### 3.3 Transient overexpression of PpINHa and PpINH3 had opposite effect on sugar accumulation in peach fruits

**Figure 3:**
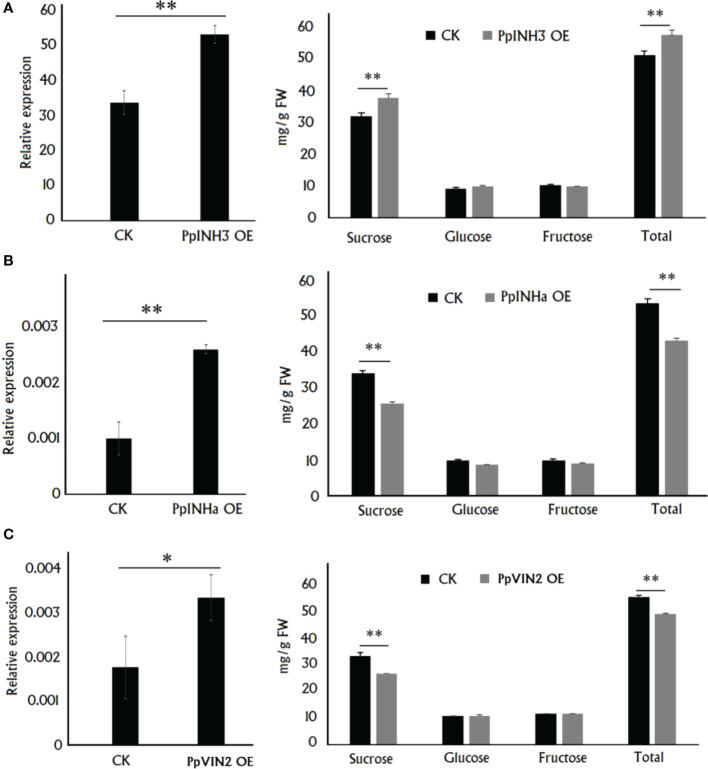
Functional analysis of *PpINHs* and *PpVIN2 via* their transient overexpression in peach fruits at the ripening stage. **(A)**, The *PpINH3* expression and sugar accumulation in the fleshy tissue surrounding the sites infiltrated with either *PpINH3* or the empty vector (CK). **(B)**, The *PpINHa* expression and sugar accumulation in the fleshy tissue surrounding the sites infiltrated with either *PpINH3* or the empty vector. **(C)**, The *PpVIN2* expression and sugar accumulation in the fleshy tissue surrounding the sites infiltrated with either *PpINH3* or the empty vector. ** and * indicate statistical significance at *P* < 0.01 and *P* < 0.05, respectively, based on Student’s *t*-test.

As mentioned above, *PpINH3* and *PpINHa* were found to act as putative positive and negative regulators of sugar accumulation, respectively. To validate this finding, we conducted functional analysis through their transient overexpression in peach fruits. Five days after transformation, gene expression level and sugar content were measured in the fleshy tissue surrounding the infiltration sites. As a result, the expression level of *PpINH3* in the flesh tissues infiltrated with *PpINH3* was significantly higher than that in the flesh tissues infiltrated with the empty vector (EV) (**Figure 3A**). The sucrose and total sugar contents in the *PpINH3-*infiltrated flesh tissues were significantly higher than those in the EV-infiltrated flesh tissues (**Figure 3A**), which is consistent the above-mentioned finding of a positive correlation between the *PpINH3* expression and the content of either total sugar or sucrose. Likewise, the expression level of *PpINHa* in the flesh tissues infiltrated with *PpINHa* was significantly higher compared to the flesh tissues infiltrated with empty vector (**Figure 3B**). However, transient overexpression of *PpINHa* resulted in a significant decrease in both sucrose and total sugar contents (**Figure 3B**), consistent with the above-mentioned finding of a negative correlation between the *PpINHa* expression and the content of either total sugar or sucrose. Altogether, these results indicated that *PpINH3* and *PpINHa* are both involved in the regulation of fruit sugar accumulation, but they have undergone functional divergence.

### 3.4 The regulatory role of PpINHa and PpINH3 in sugar accumulation is unlikely due to their interaction with PpVINs

**Figure 4:**
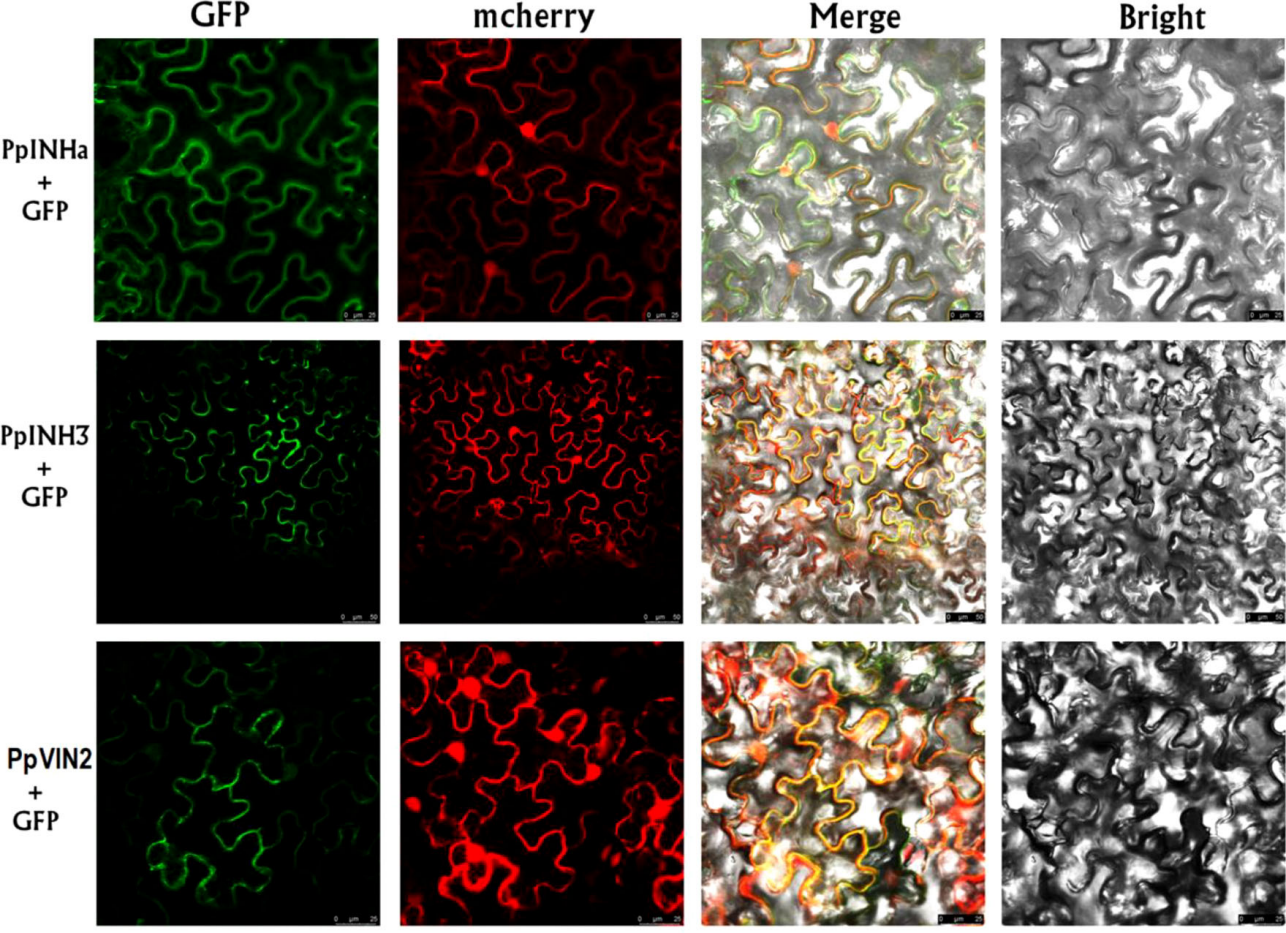
Subcellular localization of PpINHa, PpINH3 and PpVIN2 in tobacco leaves. Co-expression of psuper1300GFP-PpINHa, psuper1300GFP-PpINH3 or psuper1300GFP-PpVIN2 with tonoplast marker RFP in bright field, GFP channel, mCherry channel, and merged channel, respectively. The scale bars represent 25 or 50 µm.

Since the *INH* genes are known to function as invertase inhibitor, we investigated the interaction of *PpINHa and PpINH3* with the invertase gene. Firstly, subcellular localization assay showed that the GFP fluorescence of PpINHa-GFP or PpINH3-GFP was exactly merged with mCherry fluorescence of the tonoplast marker RFP (**Figure 4**), suggesting that PpINHa and PpINH3 were both located in the tonoplast. Therefore, we focused on whether *PpINHa* and *PpINH3* functioned as vacuolar invertase inhibitor. Our previous study showed that there are two vacuolar invertase genes *PpVIN1* and *PpVIN2* in the peach genome, and they were expressed in in immature fruits, but with extremely low or no expression in ripening fruits (Vimolmangkang et al., 2016). Similar results were also detected in this study (**Table S3**). *PpVIN2* had higher levels of expression throughout fruit development than did *PpVIN1* (**Table S3**). Therefore, *PpVIN2* was selected to investigate the interaction between PpVINs and PpINH3/a.

**Figure 5:**
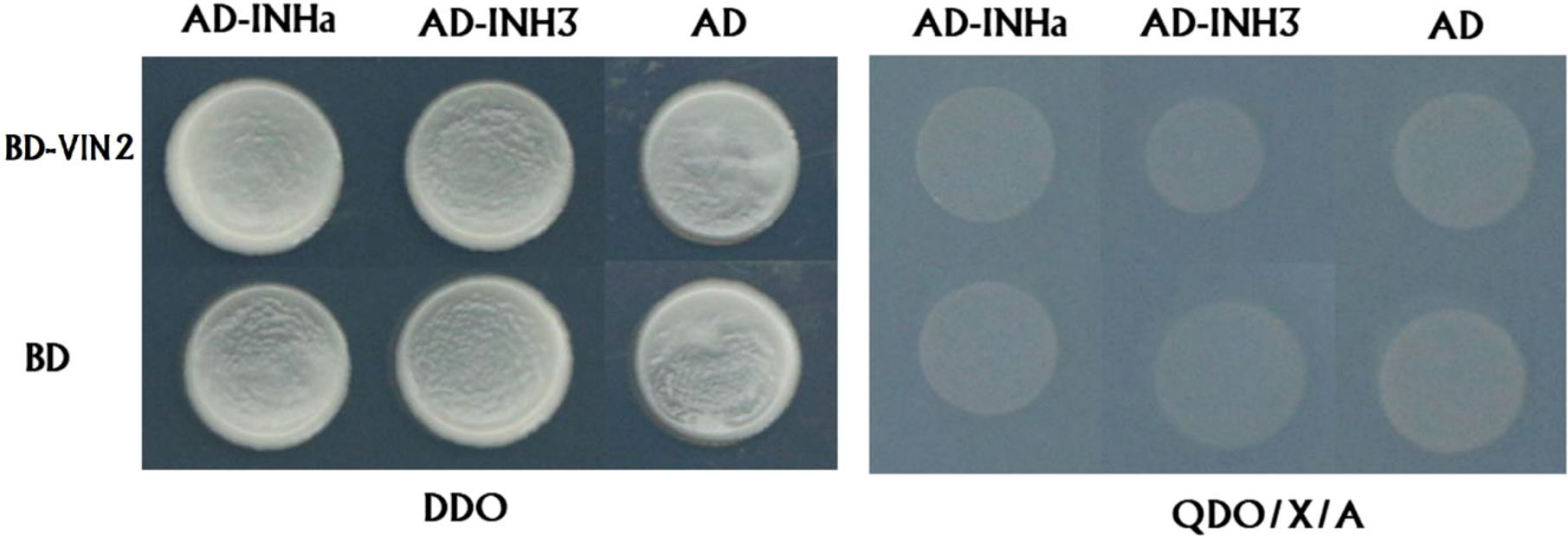
Investigation of interaction between PpVIN2 and either PpINHa or PpINH3 and using Y2H. PpVIN2 was used as bait, while PpINHa and PpINH3 were used as prey. Empty-BD and empty-AD were co-transformed as negative controls. DDO and QDO/X/A represent SD/-Leu/-Trp, SD/-Leu/-Trp/-His/-Ade/X-α-gal/AbA, respectively.

Secondly, we validated the function of *PpVIN2* using transient overexpression assay in peach fruits. Transient overexpression of *PpVIN2* in fruits of ‘Xia Huanjin’ caused a significant decrease in sucrose and total sugar contents (**Figure 3C**), suggesting its negative role in fruit sugar accumulation. Next, we conducted subcellular localization assay and the result indicated that PpVIN2, like PpINHa and PpINH3, was located in the tonoplast (**Figure 4**), suggesting the possibility of interaction between PpVIN2 and PpINH3/a. To validate the interaction of PpINHa and PpINH3 with PpVIN2, we then performed yeast two-hybrid (Y2H) assay. The coding sequence of *PpVIN2* was introduced into the bait pGBKT7 vector, while the coding regions of *PpINHa* and *PpINH3* were individually inserted into the prey pGADT7 vector. Yeast cells containing either *PpINHa* or *PpINH3* were mated with those containing *PpVIN2*. However, yeast cells containing *PpINHs* and *PpVIN2* were unable to grow on the selective QDO/X/A medium (**Figure 5**), indicating that PpINHa and PpINH3 have no interaction with PpVIN2. As mentioned above, *PpVIN1* and *PpVIN2* had no or extremely low expression in ripening fruits. These results suggested that the role of *PpINHa* and *PpINH3* in sugar accumulation could not be attributed to their interaction with *PpVINs*.

### 3.5 PpINHa and PpINH3 had an impact on transcription of genes related to fruit sugar metabolism and transport

**Figure 6:**
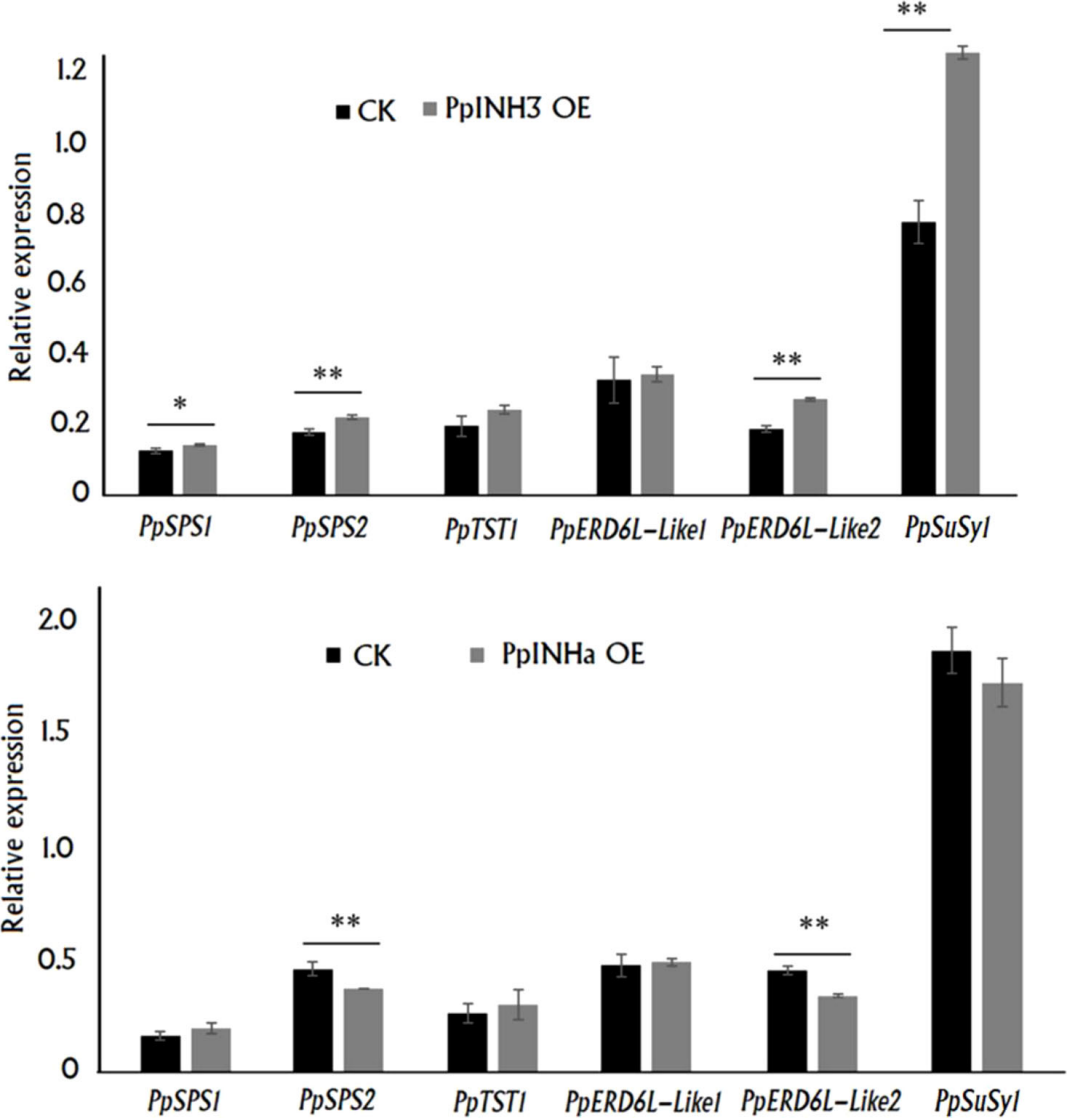
Expression of sugar accumulation-related genes in the fleshy tissue surrounding the sites infiltrated with *PpINH3, PpINHa* or the empty vector pSAK277. ** and * indicate statistical significance at P < 0.01 and P < 0.05, respectively, based on Student’s t-test.

To uncover the mechanism by which *PpINHs* regulate fruit sugar accumulation, we investigated their impact on the expression of the above mentioned DEGs associated with sugar metabolism and transport, including *PpSPS1/2*, *PpSuSy1/2*, *PpTST1* and *PpERD6-Like1/2*, in peach fruits transiently overexpressing *PpINHs* as indicated **Figure 3**. Interestingly, transient overexpression of both *PpINHa* and *PpINH3* caused a significant change in the expression of *PpSPS2* and *PpERD6-Like2* (**Figure 6**). The expression levels of *PpERD6-Like2*, *PpSPS1*, *PpSPS2* and *PpSuSy1* were significantly higher in fleshy tissues infiltrated with *PpINH3* than those in fleshy tissues infiltrated with the empty vector (CK). By contrast, the expression levels of *PpERD6-Like2* and *PpSPS2* were significantly lower in fleshy tissues infiltrated with *PpINHa* than those in fleshy tissues infiltrated with the empty vector. Notably, *PpSuSy2* showed no expression in all tested fruits. Altogether, these results suggested that *PpINHs* regulated sugar accumulation through affecting the expression of sugar metabolism and transporter genes.

### 3.6 Functional validation of sugar metabolism and transporter genes *via* transient transformation assay in peach fruits

**Figure 7:**
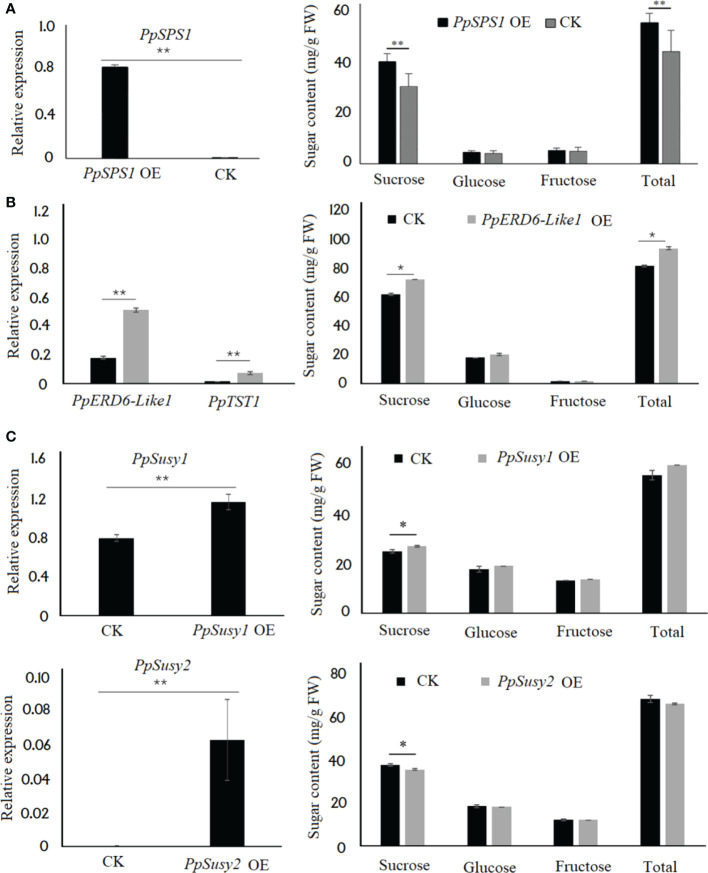
Functional analysis of sugar metabolism and transporter genes *via* transient overexpression in peach fruits at the ripening stage. **(A)**, Expression of *PpSPS1* in the fleshy tissues infiltrated with either *PpSPS1* or empty vector (CK) (left), and sugar contents in the fleshy tissues infiltrated with *PpSPS1* or empty vector (right). **(B)**, Effect of overexpression of *PpERD6-Like1* on sugar accumulation. Left, expression of *PpERD6-Like1* and *PpTST1* in the fleshy tissues infiltrated with either *PpERD6-Like1* or the empty vector. Right, sugar contents in the fleshy tissues infiltrated with *PpERD6-Like1* or empty vector. **(C)**, Effect of overexpression of the *PpSusy* genes on sugar accumulation. Left, Expression of the *PpSusy* genes in the fleshy tissues infiltrated with either *PpSusy* or empty vector. Right, sugar contents in the fleshy tissues infiltrated with *PpSusy* or empty vector. ** and * indicate statistical significance at *P* < 0.01 and *P* < 0.05, respectively, based on Student’s *t*-test.

As mentioned above, sucrose is the predominant sugar in peach fruits and the *PpSPS* genes showed a significant correlation between their expression and either total sugar content or sucrose content. Therefore, *PpSPS1* was selected to validate its role in sugar accumulation through transient overexpression in the ripening fruits of peach. Five days after transformation, the expression level of *PpSPS1* in the fleshy tissues infiltrated with *PpSPS1* was approximately 104.2 higher than that in the flesh tissues infiltrated with empty vector (**Figure 7A**). The sucrose and total sugar contents in the flesh tissues infiltrated with *PpSPS1* showed 1.32- and 1.25-fold increase, respectively, compared to the empty vector. However, no significant difference in the glucose and fructose content was detected between the flesh tissues infiltrated with *PpSPS1* and empty vector.

Since the *PpERD6-Like* genes are homologs of a previously reported *MdERDL6-1* (Zhu et al., 2021), *PpERD6-Like1* was selected to validate its impact on transcription of *PpTST1* and sugar accumulation through transient overexpression in the ripening fruits of peach. Five days after transformation, the expression levels of both *PpERD6-Like1* and *PpTST1* in the fleshy tissues infiltrated with *PpERD6-Like1* were significantly higher than in the flesh tissues infiltrated with empty vector (**Figure 7B**). Sugar content in the flesh tissues infiltrated with *PpERD6-Like1* showed a significant increase than that in the flesh tissues infiltrated with empty vector.

As mentioned above, the putative roles of *PpSuSy1* and *PpSuSy2* were different. Thus, the two *PpSuSy* genes were both subjected to functional validation using transient overexpression assay in the ripening fruits of peach. Overexpression of *PpSuSy1* resulted in a significant increase in sucrose accumulation and a slight but not significant increase in total sugar content (**Figure 7C**). By contrast, overexpression of *PpSuSy2* resulted in a significant decrease in sucrose accumulation and a slight but not significant decrease in total sugar content.

## 4 Discussion

### 4.1 Dynamic changes of soluble sugars during fruit development in peach

Soluble sugars are the important determining factor of fruit organoleptic quality in peach (Wu et al., 2014). In this study, our results indicated that soluble sugar content was consistently low in cultivated fruits during the early stages of development, but showed a dramatic increase at the ripening stage, in agreement with previous studies (Byrne et al., 1991; Desnoues et al., 2014). Unripe fruit usually accumulates high levels of starch that can serve as a temporary carbohydrate reservoir and is broken into soluble sugars during ripening, contributing to the final sugar levels (Lo Bianco and Rieger, 2002). However, peach fruit contains very low or no starch grains at the early stage of development, and starch accumulation occurs at the late stages of development (Sandhu et al., 1983; Walker et al., 2020). It is unclear whether the low content of soluble sugars during early stages of peach fruit development could be attributed to fruit growth as cell division is a complex process with high energy demands. At the ripening stage, sucrose was rapidly accumulated to become the main sugar component in fruits of three cultivars tested, which is consistent with previous findings that sucrose is the predominant component of soluble sugars in cultivated fruits (Borsani et al., 2009; Zanon et al., 2015; Monti et al., 2016; Vimolmangkang et al., 2016; Aslam et al., 2019). However, sucrose accumulation in fruits of ‘MY’ at the ripening stage showed a slight increase and sucrose only accounted for 29% of total sugars. By contrast, glucose and fructose were both dramatically accumulated to become the major sugar components, accounting for 34% and 37% of total sugars, respectively. Fructose, glucose, and sucrose are known to differ significantly in sweetness, with fructose being the sweetest carbohydrate, followed by sucrose and glucose (Ma et al., 2015). Thus, ‘MY’ could represent a valuable resource for genetic improvement of diversity of fruit organoleptic quality in peach breeding programs.

Unlike cultivated peaches, *P. davidiana* contained very low sucrose content in fruits throughout development, which is similar to the pattern of sucrose accumulation in a previously reported peach cultivar ‘Algold’ (Brooks et al., 1993). Fructose accumulated during early stages of fruit development, but decreased to trace amount during late stages of fruit development. However, glucose and sorbitol were moderately accumulated during late stages of fruit development, and sorbitol became the predominant sugar component at the ripening stage, accounting for ~ 60% of total sugars. In nectarine cultivars LG and MY, sorbitol was also dramatically accumulated during late stages of fruit development, accounting for 22% and 15% of total sugars, respectively. Sorbitol is reduced-calorie sweetener and has health benefits (Berüter et al., 1997; Fang et al., 2020). Moreover, a recent study shows that sorbitol is associated with fruit taste and shelf life (Liao et al., 2021). Hence, cultivars LG and MY as well as *P. davidiana* could be valuable resources for genetic improvement of fruit organoleptic quality and shelf life in peach.

### 4.2 Fruit sugar accumulation is controlled at the levels of biosynthesis and vacuolar storage in peach

As mentioned above, sucrose is the predominant sugar in peach fruits. SPS along with the assistance of sucrose-phosphate phosphatase (SPP) irreversibly catalyzes the formation of sucrose from glucose and fructose, while SuSy reversibly catalyzes the conversion of sucrose into glucose and fructose (Dai et al., 2013). In this study, *PpSuSy1* showed a positive correlation between its expression and either sucrose content or total sugar content, respectively, while the expression of *PpSuSy2* had a negative correlation with sucrose content and total sugar content. Transient transformation assay showed that *PpSuSy1* and *PpSuSy2* had a positive or negative role in sucrose accumulation, respectively. These results suggest that *PpSuSy1* is responsible for sucrose synthesis, while *PpSuSy2* catalyzes sucrose cleavage. Additionally, previous studies have reported a QTL on Chr7 controlling fruit sucrose content, termed qSUC.SP-G7.2 (Quilot et al., 2004) or qSUC.SZ-LG7.1_S (Desnoues et al., 2016), which is closely linked to a SSR marker pchcms2 with chromosomal position of from 18,688,514 bp to 18,688,697 bp (Vilanova et al., 2003). The physical position of *PpSuSy1* spans from 18,350,215 bp to 18,356,360 bp. Thus, *PpSuSy1* is physically close to the pchcms2 marker, suggesting that it is a good candidate for qSUC.SP-G7.2. It is worth of noting that *PpSuSy2* was weakly expressed in cultivated fruits at the ripening stage, but showed high expression in the wild fruits of *P*. *davidiana*. This may partially contribute to the difference in sugar accumulation between cultivated and wild fruits in peach. Further studies are needed to address whether *PpSuSy2* is involved in fruit sweetness selection during the domestication of peach.

Besides the *SuSy* genes, two *SPS* genes, *PpSPS1* and *PpSPS2*, both showed a significantly positive correlation between their expression and sugar content. A molecular marker ‘SPS’ on Chr1 has been identified to be associated with fruit flavor in peach (Ogundiwin et al., 2009). The ‘SPS’ maker is located in the genomic region of *PpSPS2* that spans from 40,288,490 bp to 40,295,210 bp. This QTL has been confirmed by recent studies (Li et al., 2021; da Silva Linge et al., 2021). Likewise, *PpSPS1* is located on Chr1 and its genomic region spans from 12,702,147 bp to 12,709,383 bp. The physical location of *PpSPS1* is overlapped with a previously reported QTL for fruit sugar content (Quarta et al., 2000; Quilot et al., 2004; Desnoues et al., 2016). Moreover, transient overexpression of *PpSPS1* in the ripening fruits of peach could enhance total sugar content. These results suggest that *PpSPS1* and *PpSPS2* are both strong candidate genes controlling fruit sugar accumulation in peach. Altogether, these results indicate that sucrose biosynthesis is a rate-limiting step for sugar accumulation in peach fruits.

In this study, *PpTST1* was differentially expressed between cultivated and wild fruits and its expression showed an increase during late stages of fruit development. Expression profile of *PpTST1* was in accordance with the pattern of sucrose and total sugar accumulation. These results suggest the role of *PpTST1* in regulating fruit sugar accumulation, which is consistent with previous reports that *PpTST1* is a strong candidate for QTL at the *D* locus controlling fruit sugar content (Peng et al., 2020; Yu et al., 2021). Moreover, transcription of *PpTST1* could be activated by *PpERD6-Like1* and transient overexpression of *PpERD6-Like1* could induce sugar accumulation in peach fruit. Thus, the role of *PpERD6-Like1* in sugar accumulation is different from that of previously reported *PpERDL16* that is involved in the regulation of fructose content (Yu et al., 2021). A major QTL for soluble solids content (SSC) designated qSSC.JF-ch4.1 has been identified on Chr4 in peach (Dirlewanger et al., 1999; Etienne et al., 2002; Quilot et al., 2004; Zeballos et al., 2016). The qSSC.JF-ch4.1 is located at the top of Chr4 and its closely linked SSR marker ssrPaCITA6 corresponds to the DNA fragment ranging from 2,412,721 bp to 2,412,941 bp (Dirlewanger et al., 1999). The qSSC.JF-ch4.1 is overlapped with a QTL for total sugar content that is closely linked to a RFLP marker CC129 (Quilot et al., 2004). The CC129 maker is flanked by two SNP markers SNP_IGA_382420 and SNP_IGA_382502, which have physical positions of 2,701,425 and 2,711,365 bp, respectively. The physical position of *PpERD6-Like1* spans from 2,015,166 bp to 2,019,155 bp. Given that *PpERD6-Like1* is physically close to qSSC.JF-ch4.1, it is reasonable to speculate that *PpERD6-Like1* is a good candidate for fruit sugar accumulation in peach. Altogether, these results suggest that fruit sugar accumulation is controlled at the levels of biosynthesis and vacuolar storage in peach.

### 4.3 PpINHs may play an important role in regulating fruit sucrose accumulation in peach

Vacuolar invertase responsible for sucrose cleavage in the vacuole plays an important role in the regulation of sucrose movement, storage and utilization. Here, our results showed that overexpression of *PpVIN2* caused a significant decrease in sucrose and total sugar contents, suggesting that *PpVIN2* plays a negative role in the regulation of fruit sugar accumulation. This finding is consistent with a recent report that sucrose accumulation in ripe fruit of strawberry is concomitant with the significant decrease in the expression of vacuolar invertase gene *FvVIN2* (Del Olmo et al., 2020). The activity of invertase is well known to be controlled posttranslationally through interaction with inhibitor proteins to form an inactive complex (Hothorn et al., 2004). As expected, the expression of *PpINH3* was significantly positively correlated with sugar contents. However, *PpINHa* showed a significantly negative correlation between its expression and sugar content. The opposite roles of *PpINH3* and *PpINHa* were confirmed by their transient overexpression in peach fruits. Surprisingly, the Y2H assay indicated that neither PpINH3 nor PpINHa physically interacted with PpVIN2. Thus, it is unlikely that *PpINHa* and *PpINH3* regulate sugar accumulation *via* modifying PpVINs at the posttranslational level, in accordance with the fact that the sucrose-cleaving reaction is inactive in the vacuole of sink cells of ripe peach fruit due to the extremely low expression of *PpVINs* (Vimolmangkang et al., 2016). Notably, a vacuolar invertase inhibitor gene *PpINH1* has been reported to maintain sucrose levels through inhibiting the PpVIN2 activity in peach fruit after harvest (Wang et al., 2020). Therefore, it seems that the peach *VIN* gene family is not functionally conserved.

**Figure 8:**
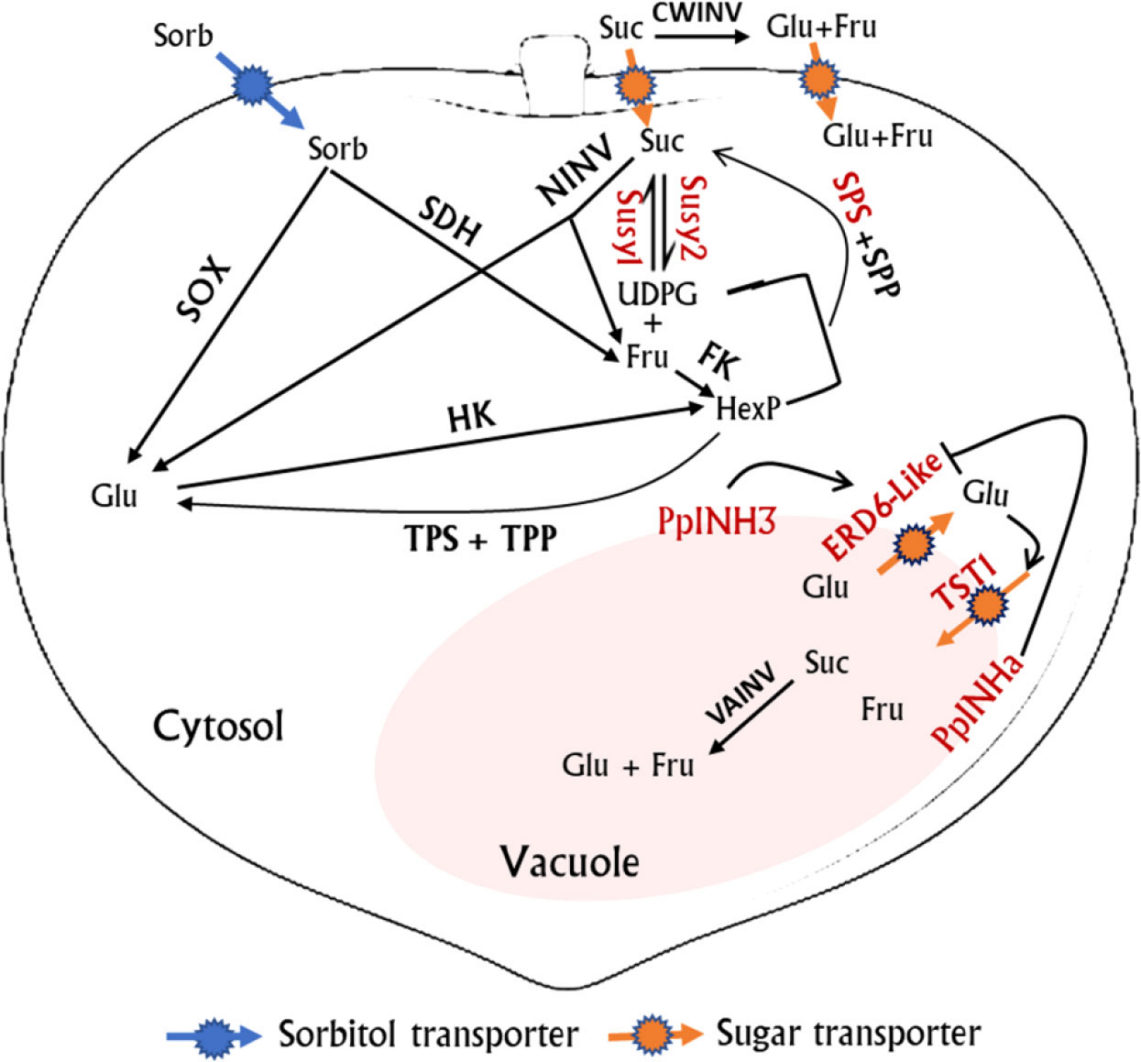
A proposed model for sugar metabolism in peach fruit. Genes whose expression levels were significantly correlated with fruit sugar content are highlighted in red color. Red streamline indicates activation. Suc, sucrose; Sorb, Sorbitol; Glu, Glucose; Fru, Fructose; UDPG, uridine diphosphate glucose; TPP, Trehalose phosphate phosphatase; TPS, trehalose phosphate synthase; and HexP, hexose phosphate.

Transient overexpression of *PpINHa* and *PpINH3* both affected the expression of *PpERD6-Like2* and *PpSPS2* in peach fruits. The activation of *PpERD6-Like2* could drive the efflux of glucose from the vacuolar into the cytosol (Zhu et al., 2021), and the increased hexose accumulation may trigger the expression of *PpSPS2*, resulting in the synthesis of sucrose and its subsequent transport to the vacuolar. Interestingly, the expression levels of *PpINH3* in ripe fruits were higher in high-sucrose cultivars XC and XH than in low-sucrose cultivars LG and MY as well as the wild relative ST, but the opposite trend was observed for *PpINHa* (**Table S3**). These results suggest an important role of *PpINHa* and *PpINH3* in the regulation of sugar content and composition. The predominant accumulation of sucrose in ripen fruits of cultivated peaches may be partially due to high *PpINH3* expression along with low *PpINHa* expression, whereas, the decreased *PpINH3* expression and the increased *PpINHa* expression are the most likely reason for the relatively low levels of sucrose in ripe fruits of ‘MY’ and ‘LG’. Based on the above results, we propose a model of fruit sugar accumulation in peach (**Figure 8**). However, further research is needed to address how *PpINHs* regulate the transcription of sugar metabolism and transporter genes, and thus affecting sugar accumulation.

## Data availability statement

The original contributions presented in the study are publicly available. This data can be found here: NCBI, PRJNA626460 and PRJNA787711.

## Author contributions

LW and YH planned and designed the experiments. LZ collected experimental materials. MM, XZ, XJ, QY, YC SC and LW performed data analysis. MM, XZ, EN and QP performed the experiments. MM, LW and YH wrote the manuscript. YH revised the manuscript. All authors contributed to the article and approved the submitted version.
