# Supplementary material for: Two vacuolar invertase inhibitors PpINHa and PpINH3 display opposite effects on fruit sugar accumulation in peach
Source: Front Plant Sci. 2022 Dec 14;13:1033805. doi: 10.3389/fpls.2022.1033805 (PMC9795002; doi:10.3389/fpls.2022.1033805)
Supplement: Supplementary file 1 [file DataSheet_1.docx]

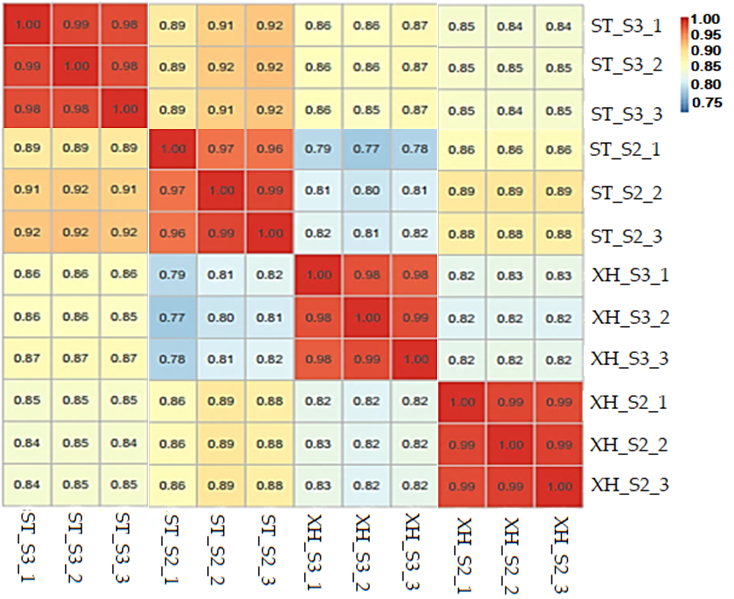


Figure S1. Correlation of gene expression profiles between different libraries.


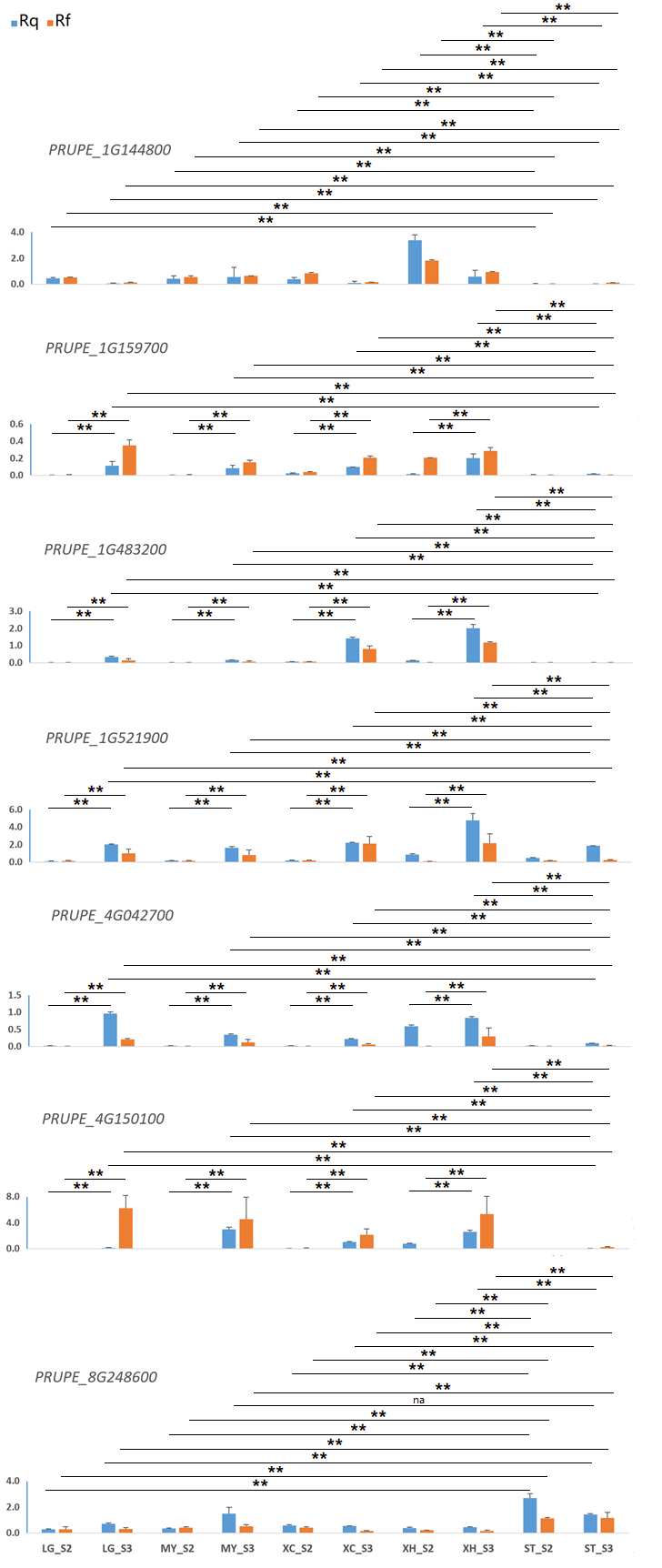


Figure S2. Validation of DEGs using quantitative real-time PCR (qRT-PCR). The relative expression levels of selected nine genes in two developmental stages of four cultivars and the wild relative ST based on the FPKM value (Rf) and qRT-PCR (Rq). Bars show mean ± SE (n = 3).


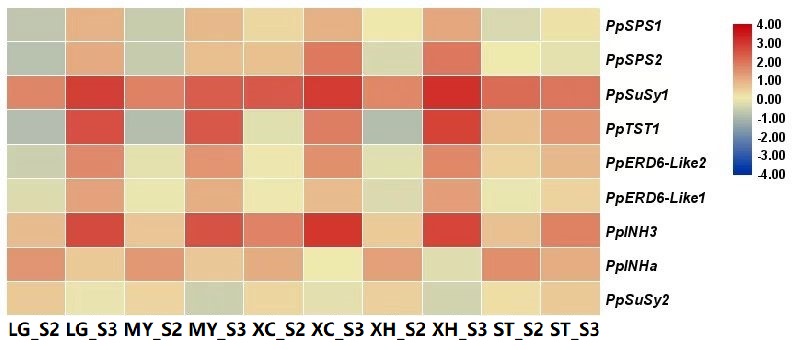


**Figure S3.** Heatmap of transporter-related DEGs that were potentially associated with fruit sugar accumulation.
